# The Book World of Medicine and Science

**Published:** 1904-01-16

**Authors:** 


### The Book World of Medicine and Science.

Tuberculosis of the Female Genitalia and Peritoneum. By John B. Murphy, O.M., M.D., Chicago. P. 119.

Tuberculosis of the organs of generation in the female has of late years attracted very considerable attention, both in its pathological and in its clinical aspect. Dr. Murphy has written a very complete review of the present state of knowledge of this subject in Germany and America, but following the almost universal custom of American authors, he fails to give to English observers the credit to which their researches entitle them. The opening sections of the address are devoted to the history, frequency, and etiology of the condition. It is interesting to note that it was first recognised by Morgagni in 1744, and, as would be expected, the first case investigated was one of tuberculous salpingitis. The incidence is difficult to gauge. Probably about 10 per cent. of women who die from tuberculosis present lesions in some part of the genital tract, and far the most frequent site is the fallopian tube. Primary infection of these organs is a much rarer occurrence, but many well-marked cases are cited. The mode of infection is discussed at some length, and the results of the author's own investigations upon monkeys are of great interest; the conclusion arrived at is that Hegar's distinction between infections of an ascending and of a descending nature still holds good. In the second part of the address infections of the vulva, vagina, cervix, body, fallopian tube and ovary are considered in detail. Many cases are cited, the signs and symptoms are summarised and the treatment is discussed. Of special interest is the section upon tuberculosis of the cervix, Lewer's classical case is quoted, but there is no mention of the very interesting cases of Croft and of Brooke reported some months ago in the "Journal of Obstetrics of the British Empire." The third part consists of an essay upon tuberculous peritonitis, and the treatment is dealt with in great detail. Four varieties are recognised: the exudative, the ulcerative, the adhesive and the cases of mixed infections. The pros and cons for operative treatment in each of these varieties are discussed with admirable clearness. Dr. Murphy's book will well repay perusal; it is a careful and thorough piece of work, and is especially valuable as embodying the opinions of an operative surgeon of great experience.

International Clinics. Vol. III. Thirteenth Series. By various authors. Edited by A. O. J. Kelly, M.D., Philadelphia. (London: J. B. Lippincott Company. Pp. 305. Price 10s. 6d. net.)

We have had very much pleasure in reading previous volumes of these "International Clinics," and we think, with regard to the book before us, that it would be difficult to offer any better praise than to say it is quite up to the high level of its predecessors. The work fully maintains its cosmopolitan character. Under the heading of Diseases of the Gall-bladder and Gall-ducts papers are contributed by John H. Musser, M.D.; R. D. Rudolf, M.D.Edin., M.R.C.P. Lond.; Charles G. Stockton, M.D.; F. Parkes Weber, M.D., F.R.C.P.; F. Sejars, M.D., and John B. Deaver, M.D. Collectively and individually these papers form an extremely valuable addition to the literature of medicine. David W. Finlay, M.D., F.R.C.P., contributes an article on the Treatment of Pneumonia; Albert Robin, M.D., one on the Medical Treatment of Gastric Cancer; Achilles Rose, M.D., discusses the carbonic acid treatment in rectal diseases; and A. Chantemesse deals with the Serum Treatment of Typhoid Fever. Under the section of Medicine papers are contributed by Charles F. Craig, M.D.; R. Murray Leslie, M.B., M.R.C.P.; Thomas J. Mays, M.D; J. S. Fowler, M.D., F.R.C.P.Edin.; and F. J. Poynton, M.D., F.R.C.P. Various surgical subjects are dealt with by J. A. Bodine, M.D.; John A. Lewis, M.D.; Professor Lucas-Championnière; William L. Rodman, M.D.; William T. Belfield, M.D.; and C. E. Schwartz, M.D.

A glance at the names of these contributors will be a sufficient guarantee of the excellence of the material contained in this volume. We cannot do better than wish these "clinics" a continued prosperity, and heartily recommend them to the attention of everyone engaged in the study and practice of medicine.

A Pocket Book of Clinical Methods. By C. H. Melland, M.D.Lond., M.R.C.P. Pp. 88. (Bristol: John Wright and Co. 1903.)

This little book, Dr. Melland states in the preface, has been prepared with the main object that it should serve as a guide to the details of methods of clinical laboratory examination, which are adapted to the use of students. In his laudable endeavour to restrict the scope of the work within the limits he has imposed, the author has not always been quite happy in his decisions concerning what to include and what to omit. For example, in the section devoted to the examination of the fæces, details are given as to how to search for the amœba dysenteriæ and tubercle bacilli, but no help is given towards the important matter of examining the fæces for fat, undigested muscle fibres, or small traces of blood. However, this is the only fault we can find, and both the material given and the manner of setting it forth are excellent. There is an index which adds considerably to the value of the book. On the whole, Dr. Melland is to be congratulated on producing a really useful little volume.

Medical and Surgical Reports of the Boston City Hospital, 1903. Fourteenth Series. Edited by H. L. Burrell, M.D., W. T. Councilman, M.D., and C. F. Withington, M.D.

This publication consists of a series of papers on medical and surgical subjects by members of the staff of the Boston City Hospital. The articles are of a high standard, and although it may seem invidious to make distinctions where all is so good, we venture to mention the article on Relapse in Pneumonia by A. Lawrence Mason, M.D., and that on Movable Kidney by Ralph C. Larrabee, M.D., as being especially valuable. These Reports, like many other so-called medical and surgical reports, contain no statistics or tables, and beyond incidental references to particular cases and specimens they do not give a survey of, or deal in a general manner with the surgical and medical work of the Boston City Hospital; the title of the volume is therefore somewhat of a misnomer.

Ethyl Chloride in Surgical and Dental Practice. By A. de Prenderville, M.R.C.S., L.R.C.P. (London: Henry J. Glaisher. 1903. Price 1s. net.)

In this little pamphlet the author sets forth the advantages which may be claimed for ethyl chloride as an anæsthetic, whether used by itself for operations of short duration or as a preliminary to the administration of ether or chloroform. Among other points in its favour are mentioned the rapidity with which anæsthesia ensues, the absence of struggling in the preliminary stages, the possibility of keeping the patient in the sitting posture during operations on the nose and mouth, the absence of cyanosis and congestion, and the consequent lessening of hæmorrhage. The author also describes a contrivance by which ethyl chloride may be administered with an ordinary gas and ether apparatus.

